# Positive Seatbelt Sign with Avulsed Leiomyoma following Motor Vehicle Accident Leading to Hemoperitoneum

**DOI:** 10.1155/2018/4251408

**Published:** 2018-08-26

**Authors:** Martin A. C. Manoukian, Amode R. Tembhekar, Sarah E. Medeiros

**Affiliations:** ^1^University of California Davis School of Medicine, 4610 X Street, Sacramento, CA 95817, USA; ^2^Department of Emergency Medicine, University of California, Davis, 2315 Stockton Boulevard, Sacramento, CA 95817, USA

## Abstract

A positive seatbelt sign following a motor vehicle accident is associated with an increased risk of intra-abdominal injury and hemoperitoneum. Injury to the uterus in reproductive-age women can also occur. In this report, we describe a 29-year-old nulligravida female who presented to the emergency room following a motor vehicle accident at freeway speeds. A positive seatbelt sign was noted, and a focused assessment with sonography for trauma revealed hemoperitoneum with an incidental finding of uterine leiomyomata. Upon exploratory laparotomy, a free-floating intraperitoneal mass was identified as an avulsed uterine leiomyoma. A uterine laceration containing a subserosal leiomyoma was also identified. The gynecological team was consulted, and a myomectomy of the subserosal leiomyoma followed by a closure of the uterine laceration was performed. The patient was transfused with a total of three units of packed red blood cells and two units of fresh frozen plasma. The postoperative course was without major complication. A positive seatbelt sign and hemoperitoneum in a reproductive-age woman with leiomyomata should increase the clinical suspicion for uterine injury and decrease the threshold for obtaining a gynecological consultation.

## 1. Introduction

Abdominal injuries resulting from motor vehicle accidents (MVAs) make up a significant number of emergency room visits every year. A rapid evaluation by a trained medical team that is able to determine the severity of injury is imperative in the quick and appropriate treatment of critically injured patients. Due to the rising use of vehicular restraints in the United States, the incidence of a positive “seatbelt sign,” a band of ecchymosis on the patient's abdomen, following an MVA is increasing [1, 2]. The presence of a seatbelt sign raises the clinical suspicion for hemoperitoneum, which can lead to a rapid loss of intravascular blood and hypoxic end organ damage. In addition to a positive seatbelt sign, physical exam findings suggestive of hemoperitoneum include rebound tenderness, hypotension, and abdominal distension; however, a bedside FAST exam is the most accurate assessment tool [3]. Common causes of hemoperitoneum seen alongside a seatbelt sign include injury to the bowel, spleen, liver, or major vasculature [4]. However, damage to pelvic organs must also be considered. Herein, we report a case of a patient who presented to our emergency department (ED) with a positive seatbelt sign and hemoperitoneum of pelvic origin due to an MVA.

## 2. Case

**Figure 1:**
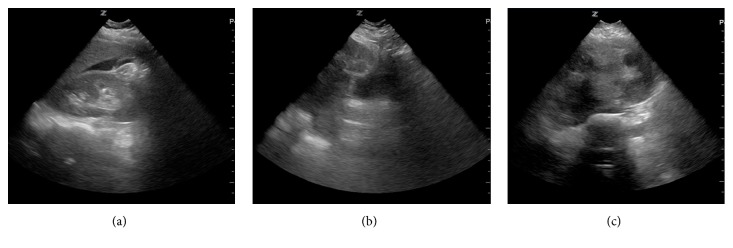
Free fluid noted in right upper quadrant on FAST scan (a). Incidental leiomyomata uteri noted as seen in the sagittal (b) and transverse (c) planes.

**Figure 2:**
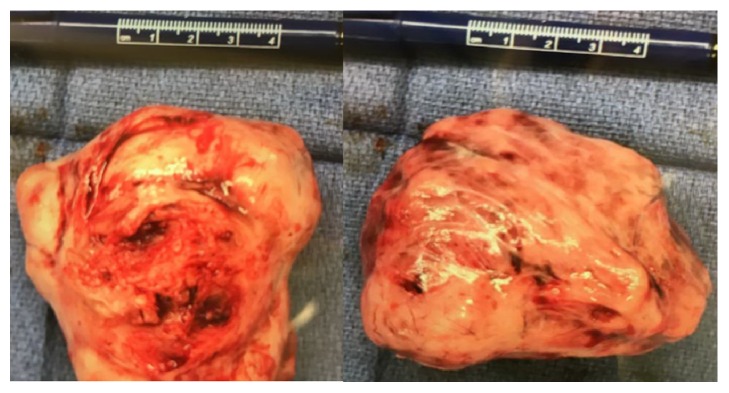
Two images of the 6 cm x 8 cm avulsed uterine leiomyoma recovered from the patient during laparotomy.

A 29-year-old G0P0 South Asian female was brought into the ED via ambulance following an MVA at freeway speeds involving multiple vehicles. The crash resulted in the deployment of the airbags and a subsequent loss of consciousness in the patient lasting less than one hour. Blood pressure was 89/40 on scene and improved to 117/95 en route to the ED. Upon arrival to the ED, the patient complained of 10/10 pain in the abdomen and left hip. Triage vitals were as follows: blood pressure 96/58, heart rate 85 beats/minute, respiratory rate 19 breaths/minute, and Glasgow coma scale 15/15. The patient arrived with a cervical collar and backboard in place and was noted to have a positive seat belt sign. A focused assessment with sonography for trauma (FAST) exam was positive in the right upper quadrant (Figure 1(a)). Leiomyomata uteri were incidentally noticed on ultrasound (Figures 1(b) and 1(c)). A pelvic X-ray showed no acute fracture or traumatic malalignment. Hemoglobin was 10.7 grams/deciliter (reference range 12-16 grams/deciliter). The patient was subsequently taken to the operating room where a midline laparotomy was performed with an immediate upwelling of blood. The abdomen was packed in all four quadrants to control bleeding and stabilize blood pressure. Upon unpacking and inspection of the upper quadrants, no damage was observed to the mesentery, colon, liver, or spleen. Inspection of the lower quadrants revealed a free-floating mass of tissue later identified as a leiomyoma (Figure 2). In addition, the uterus was noted to be bleeding from a 5 cm fundal laceration. The uterus appeared fibroid in character, and a 3 cm subserosal leiomyoma was seen extending into the laceration. The gynecologic team was consulted and proceeded to inject 20 units of vasopressin in 60 cc dilution into the uterus. This was followed by a myomectomy of the subserosal leiomyoma and closure of the uterine laceration. The abdominal incision was then closed, with a total estimated blood loss of 1000 mL. The patient was administered 2 units each of red blood cells and fresh frozen plasma, as well as 1.3L of crystalloid intraoperatively. Hemoglobin on post op day 1 downtrended to 6.5, at which point a third unit of red blood cells was given. On post op day 2, the patient developed intractable nausea, after which a nasogastric tube was inserted and 1 liter of green bilious output was achieved. The patient was stabilized and transferred to an outside hospital on post op day 3 for further recovery.

## 3. Discussion

It is often difficult for an ED physician to determine the seriousness of an MVA from the quick report delivered by emergency medical services upon patient arrival. Frequently, short phrases such as “accident occurred at freeway speeds” or “vehicle rolled multiple times” are the only summaries provided before information on the patients' vitals and ambulance course is communicated. One concept that may assist emergency physicians in determining the severity of an MVA is that of energy equivalent speed (EES), a measure of the kinetic energy that is dissipated during a vehicular accident [5]. Though many variables contribute to the calculation of EES, simply put, EES is directly proportional to the change in velocity of the vehicle in which the patient is traveling and indirectly proportional to the mass of the vehicle. Thus, greater changes in velocity and vehicles with lower mass yield higher EES. By inquiring as to the speed of the vehicle, the postimpact trajectory of the vehicle (immediate stop after striking a wall versus a more gradual deceleration) and mass of the vehicle in which the patient was traveling, an ED physician may be able to better anticipate severe injury to the patient. Higher EES has been linked to increased injury severity as well as increased mortality, with most mortalities occurring due to either polytrauma or hemorrhage [6, 7]. A report from emergency medical services suggesting a high EES combined with a seat belt sign on initial exam should raise a physician's index of suspicion for serious intra-abdominal injury.

Though hemoperitoneum following MVAs typically originates from abdominal organs, in reproductive-age women other important life-threatening etiologies must be considered and ruled out. In pregnant women, seatbelt sign injury can lead to fetal injury or demise due to placental ischemia, abruptio placentae, or uterine damage. However, it should be noted that a properly worn seatbelt does not increase perinatal injury following MVA [2, 8]. Uterine damage must also be considered in nongravid women. Uterine enlargement secondary to leiomyomas or adenomyosis can increase its susceptibility to damage in an MVA. Uterine leiomyomas are thought to occur in up to 25% of women, and previous case reports have shown that traumatic abdominal injury following an MVA can lead to an avulsion of a uterine leiomyoma, resulting in hemoperitoneum with a free-floating intraperitoneal mass [9–12]. However, none of these reports include a positive seatbelt sign, which is traditionally associated with intra-abdominal injury. Additionally pelvic radiographs in the aforementioned case reports either indicated the presence pelvic fracture or are not commented upon. In contrast, the patient in this report had both a positive seatbelt sign and an unfractured pelvis. Thus, it can be inferred that the avulsed leiomyoma originally extended superiorly from the uterus to a point above the pubic ramus where it was exposed to trauma from the seat belt. This led to its avulsion and subsequent hemoperitoneum in the patient. Though less likely, traumatic hemoperitoneum with a free-floating mass can also be due to rupture of a cystic teratoma (this case report also lacks a positive seat belt sign) [13]. Other possible etiologies of a free-floating intraperitoneal mass in women can include an ovary, gallbladder, or mesothelial cyst [14, 15].

Although no clear diagnostic tool to confirm gynecologic injury in MVA induced abdominal trauma exists, several clinical signs can be used to raise suspicion. First, a seatbelt sign with lower abdominal ecchymosis in reproductive-age women may indicate uterine injury, even in the absence of pelvic fracture or malalignment on radiographic imaging as seen in this patient. Secondly, incidental uterine enlargement on FAST exam, also seen in this patient, can help the emergency medicine and trauma teams be conscious of possible uterine involvement and thus alert the gynecological team. Though many trauma surgeons are capable of operating on pelvic organs, gynecological surgeons have more experience in uterine specific procedures, such as the myomectomy that was performed on this patient, and may achieve better outcomes. Lastly, the detection of a free-floating intraperitoneal mass in reproductive-age women should immediately warrant consideration of gynecologic injury and consultation of the gynecological surgery team. Through the identification of these factors, emergency medical and trauma surgery teams may more quickly identify a gynecological source of hemoperitoneum. This could result in a more rapid consultation of gynecological surgeons, decreasing the time to treatment and blood loss, thereby improving outcomes.

## References

[1] Glover J. M., Waychoff M. F., Casmaer M. (2017). Association between seatbelt sign and internal injuries in the contemporary airbag era: A retrospective cohort study. *The American Journal of Emergency Medicine*.

[2] Masudi T., McMahon H. C., Scott J. L., Lockey A. S. (2017). Seat belt-related injuries: A surgical perspective. *Journal of Emergencies, Trauma, and Shock*.

[3] Nishijima D. K. (2012). Does this adult patient have a blunt intra-abdominal injury?. *JAMA*.

[4] Biswas S., Adileh M., Almogy G., Bala M. (2014). Abdominal injury patterns in patients with seatbelt signs requiring laparotomy. *Journal of Emergencies, Trauma, and Shock*.

[5] Berg F. A. Implications of Velocity Change Delta-V and Energy Equivalent Speed (EES) For Injury Mechanism Assessment in Various Collision Configurations.

[6] Miltner E., Salwender H.-J. (1995). Injury severity of restrained front seat occupants in car-to-car side impacts. *Accident Analysis & Prevention*.

[7] Miltner E., Salwender H.-J. (1995). Influencing factors on the injury severity of restrained front seat occupants in car-to-car head-on collisions. *Accident Analysis & Prevention*.

[8] Yamada S., Nishijima K., Takahashi J., Takahashi N., Tamamura C., Yoshida Y. (2017). Intrauterine fetal death caused by seatbelt injury. *Taiwanese Journal of Obstetrics and Gynecology*.

[9] Sparic R. (2016). Epidemiology of Uterine Myomas: A Review. *International Journal of Fertility & Sterility*.

[10] Venables C. W., Craft I. L. (1967). Haemoperitoneum after Traumatic Avulsion of Uterine Fibroid. *British Medical Journal*.

[11] Seiji M., Shinnichi I., Motojyuku M., Akieda K., Yamamoto I., Inokuchi S. (2005). Traumatic avulsion of the uterine myoma. *Journal of Trauma - Injury Infection and Critical Care*.

[12] Hicks C. W., Garcia L., Howley I. (2014). Traumatic hemoperitoneum. *JAMA Surgery*.

[13] Levine R. L., Pepe P. E., Blackstone W., Danzinger J., Varon J. (1992). Occult traumatic avulsion of an ovarian dermoid cyst. *The American Journal of Emergency Medicine*.

[14] Uygun I., Aydogdu B., Okur M. H., Otcu S. (2012). The First Report of an Intraperitoneal Free-Floating Mass (an Autoamputated Ovary) Causing an Acute Abdomen in a Child. *Case Reports in Surgery*.

[15] Watson H. I., Borovickova M., Shetty A. (2014). The curious case of free-floating pelvic cysts. *BMJ Case Reports*.

